# *Vibrio* spp and other potential pathogenic bacteria associated to microfibers in the North-Western Mediterranean Sea

**DOI:** 10.1371/journal.pone.0275284

**Published:** 2022-11-30

**Authors:** Maria Luiza Pedrotti, Ana Luzia de Figueiredo Lacerda, Stephanie Petit, Jean François Ghiglione, Gabriel Gorsky

**Affiliations:** 1 Laboratoire d’Océanographie de Villefranche sur mer (LOV), UPMC Université Paris 06, CNRS UMR 7093, Sorbonne Université, Villefranche sur Mer, France; 2 Laboratoire d’Océanographie Microbienne, UMR 7621, Observatoire Océanologique de Banyuls, Sorbonne Université, CNRS, Banyuls-sur-Mer, France; Duke University Marine Laboratory, UNITED STATES

## Abstract

Microfibers, whether synthetic or natural, have increased dramatically in the environment, becoming the most common type of particles in the ocean, and exposing aquatic organisms to multiple negative impacts. Using an approach combining morphology (scanning electron microscopy-SEM) and molecular taxonomy (High-Throughput DNA Sequencing- HTS), we investigated the bacterial composition from floating microfibers (MFs) collected in the northwestern Mediterranean Sea. The average number of bacteria in 100 μm^2^ on the surface of a fiber is 8 ± 5.9 cells; by extrapolating it to a whole fiber, this represents 2663 ± 1981 bacteria/fiber. Attached bacterial communities were dominated by Alteromonadales, Rhodobacterales, and Vibrionales, including the potentially human/animal pathogen *Vibrio parahaemolyticus*. This study reveals a high rate of bacterial colonization on MFs, and shows that these particles can host numerous bacterial species, including putative pathogens. Even if we cannot confirm its pathogenicity based only on the taxonomy, this is the first description of such pathogenic *Vibrio* living attached to MFs in the Mediterranean Sea. The identification of MFs colonizers is valuable in assessing health risks, as their presence can be a threat to bathing and seafood consumption. Considering that MFs can serve as vector for potentially pathogenic microorganisms and other pollutants throughout the ocean, this type of pollution can have both ecological and economic consequences.

## 1. Introduction

Plastics are synthetic organic polymers whose mass production began in the 1950s, and has grown from 1.5 million tons/year to 359 million tons in 2018 [1], with cumulative global production expected to triple by 2050 to 33 billion tons [2]. Due to their versatility, low production cost, and resistance to degradation, plastic became a key product in our society; however, its low degradation rate has become an environmental threat [3, 4]. Plastics are the most abundant contributors to marine litter (60–90%), and in their majority they consist of microplastics (< 5 mm in size) [5, 6]. It is currently estimated that 4.8 to 12.7 Mt of plastic enter the ocean each year [7], with floating plastic concentrations between 1–5 millimeters accounting for 24.4 trillion items, weighing between 82,000 and 578,000 tons [8]. Despite current strategies to reduce plastic pollution, the projected growth in plastic production, and thus plastic waste, exceeds efforts to mitigate plastic pollution [9].

The textile industry is of great economic value where more than half of the world’s production is based on synthetic fibers, with polyester, polyamide, acrylic and polyolefin being the most common types, the rest of the production comes from natural fibers with cotton being the most important [10]. Synthetic microfibers are a ubiquitous class of microplastics that can have several origins: they come from household laundry textiles that enter the oceans via urban wastewater treatment plants (WWTPs) or rivers that carry plastic waste from inland [11, 12], through atmospheric deposition or aerosols [13–15], as well as derived from fishing activities [16].

Microfiber pollution is widespread in coastal and offshore surface waters in all ocean basins; synthetic fibers account for up to 50% of fibrous items; the remainder are natural fibers such as cotton and wool [17–19]. Whether synthetic or natural polymers, their release into the environment has become an emerging pollution concern because organisms are exposed to this mixture on a daily basis, and little is known about the degradation of MFs in the marine environment and how it can pose a potential long-term risk to ecosystems and human health [20, 21]. In addition, synthetic microfibers are now the most common type of anthropogenic particles in the oceans, in some cases accounting for 80–90% of the number of microplastics, with higher concentrations than granules or fragments [22]. Their release into the environment poses a threat to marine ecosystems, as they are the most common type of ingested microplastics [23, 24]. They may be potentially harmful physically and chemically for aquatic environments or the food chain through the release of additives and dyes, with unknown consequences, including for humans [25–27].

Once at the sea, like other microplastics, the fibrous material can be rapidly colonized by microorganisms, such as bacteria or benthic microalgae [28]. The establishment of a biofilm on microplastics, which can appear and smell like food [29] may enhance their ingestion. Several studies have shown that bacterial communities inhabiting plastic on the surface of the oceans differ significantly from bacterial communities in the surrounding water [30–33]. Plastics can also become vectors for potentially dangerous or pathogenic microorganisms, and their impact on marine environments and human health is the subject of numerous studies [34–37].

The Mediterranean is a semi-enclosed sea with a densely populated coastline and high economic activity, tourism, and maritime traffic that result in a significant land-based plastic pollution, which represents over 80% of the marine litter [6, 38]. This sea, although it represents only 0.8% of the world’s marine waters, was predicted by global models to account for nearly 7% of microplastic pollution in the global ocean [6]. The Mediterranean also hosts a high level of biodiversity with 17,000 marine species, 28% of which are endemic [39], that are likely to be affected by the presence of plastic debris in all marine compartments [40].

Here we examined the interaction between MFs and the microbial community of the Mediterranean Sea, with focus on Vibrionales. We also discussed the potential role of these fibrous particles, now ubiquitous in the marine environment, as vectors for the spread of potential pathogens such as *Vibrio parahaemolyticus*. This study is part of a larger survey where general information on the abundance of MFs, as well as their chemical composition in the Mediterranean Sea is published in Pedrotti and colleagues [19]; they found, in the same samples we used to study the organisms attached to MFs, that 14–50% of MFs are synthetic fibers. The term MFs used in this study therefore refers to all types of fibers (synthetic or natural).

## 2. Material and methods

### 2.1 Sampling collection

**Fig 1:**
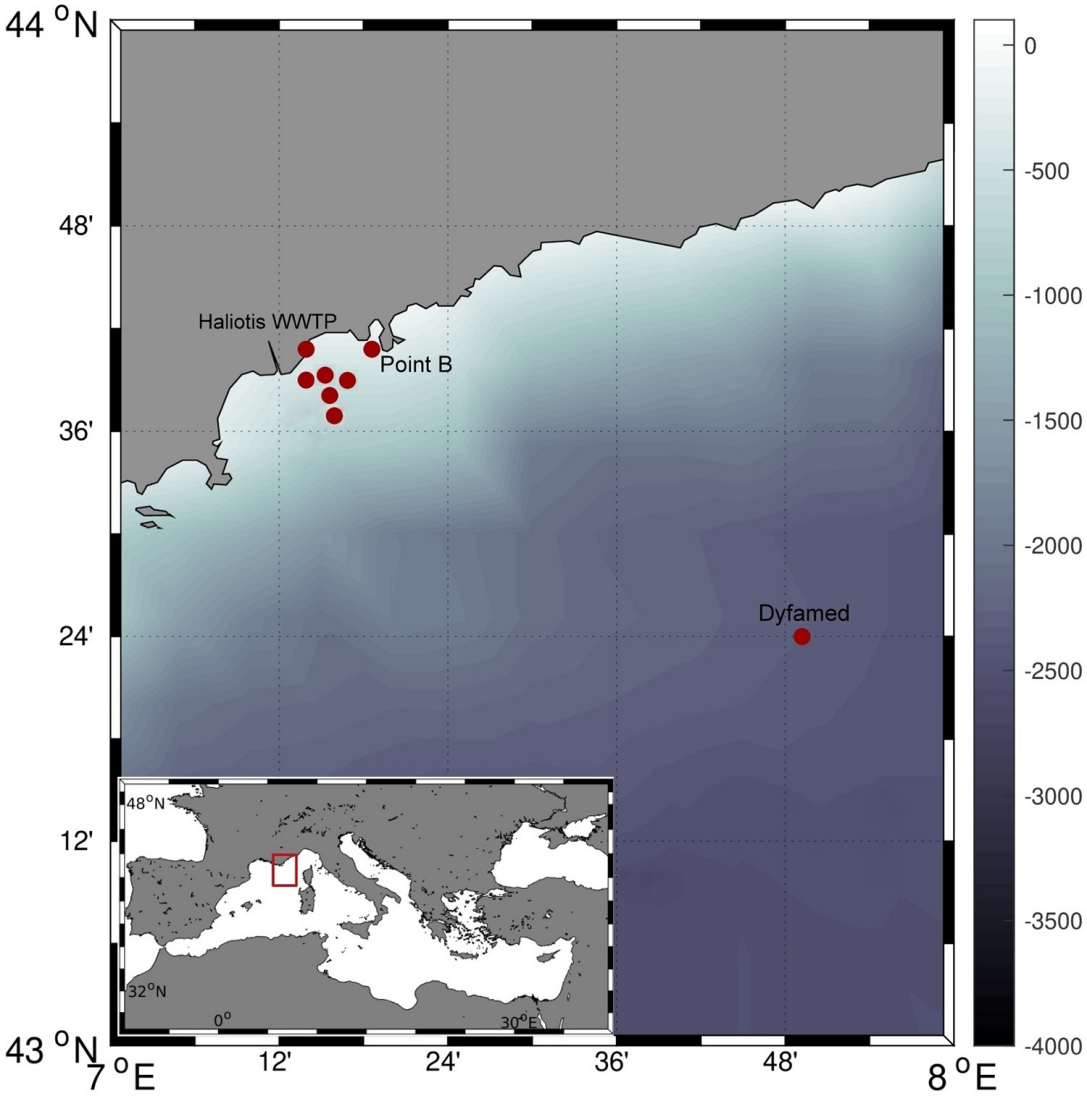
Map of the northwestern Mediterranean Sea where seasonal sampling of MFs and seawater was carried out in the coastal area between Nice and Monaco, as well as in the offshore Dyfamed station, and the Haliotis waste water treatment plant. This map was created at MATLAB software by using the *M_Map* package.

Samples were collected aboard the research vessel The Alchemy, an 11 m long sailing ship, in the northwestern Mediterranean Sea, Liguria Sea, during the ECOSEASTEM cruise (February—October 2014) [19]. For this experiment, a total of seven sites were sampled in spring (6–8 May) and summer (5–8 August). Six sites were located along the coastal zone from the Var River, from in front the city of Nice (where the Haliotis waste water treatment plant—WWTP—is located) to the entrance of the bay of Villefranche-sur-mer (Point B). Another sampling station, characterized by low anthropogenic contamination, was located 70 km off the coast (DYFAMED station). Samples were also collected from the urban and treated water of the Nice Haliotis WWTP (Fig 1).

For *in situ* samples, fifty liters of surface seawater were collected with a stainless-steel bucket in each location and filtered through a 20 μm pore sized stainless-steel sieve. Samples were then resuspended in 200 mL of 0.2 μm filtered seawater. A quarter of each sample was used to investigate the microbial diversity associated to MFs by DNA analysis, and another quarter was destined for image analysis of MFs-attached biota. The remaining subsamples were dedicated to the quantification and characterization of MFs by Fourier transform infrared spectroscopy (ATR-FTIR) in a larger experiment, provided in Pedrotti and colleagues [19].

In parallel, 2 L of surface seawater were sampled to analyze the free-living microbial community. For sampling in the WWTP, three replicates of 2 L of urban and treated wastewater were also collected during the two seasons. All water samples were collected in amber bottles and fixed in 2% formaldehyde.

To minimize contamination, a stainless-steel bucket, held by sheathed steel wire, was used for collecting surface water and, at each sampling point, it was rinsed three times with distilled water. Sampling was carried by placing the bucket on the surface of the water, so that only the top 10 cm was sampled. To avoid cross-contamination, in both in situ sampling and laboratorial manipulation, all material was previously washed with alkaline and acidic detergents, and rinsed three times before each handling with 0.2 μm milli-Q microfiltered water.

### 2.2 Scanning electron microscopy (SEM) & image analysis

SEM images were obtained to quantify and evaluate the morphology of microbial organisms on MFs. Seawater was filtered through 0.2μm-pore-size Anodisc membrane (aluminumoxide, 47mm). The filters were dehydrated with alcohol (1 rinsing for 10 min at 70%, rinsing 10 min at 96% and 3 rinses 10 minutes with absolute ethanol) and fixed under a chemical hood with Hexamethyldislazan (HDMS). Analysis was performed with a JEOL6700F field emission gun SEM equipped with a cryo-fracture platinum Gryan Alto 2500 and a YAG Autrata backscattered electron detector (X25-X20 000). Prior the analyzes, the fiber length was measured using a stereomicroscope (Zeiss Discovery V12 SZX10) and ImageJ v.1.5 software, and benchmarks were sheared on the filter to first identify the MFs to allow their identification under a scanning microscope. The number of bacteria attached to the fibers was calculated based on the SEM images. For this, bacteria were counted in several fields of 30 MFs using ImageJ v.1.5 software. In order to report the bacterial abundance on the surface of a fiber, we considered it as a cylinder without the spherical ends. Considering the median length (601 μm) and the median diameter (18μm) of the fibers analyzed, this represented an average scanned area of 33986 μm^2^.

### 2.3 Samples processing prior DNA extraction

In the laboratory, the resuspended sieved sample represented the community associated with the MFs (MF fraction). To study the microbial diversity of free-living community, the seawater was filtered through 0.2μm-pore-size filters (47 mm diameter, polycarbonate, Nuclepore). For wastewater, 100 ml of inlet samples and 300 ml of outlet samples were resuspended through the 20μm sieve, and filtered through 0.2μm-pore-size filters (47 mm diameter, polycarbonate, Nuclepore). Filters were stored at -80°C until DNA extraction.

### 2.4 DNA extraction, PCR and high-throughput sequencing

DNA extraction was performed using a thermo-saline lysis protocol [41]. The partial 16S rRNA gene was amplified by PCR using primers 357F and 907R, which amplify the V3-V5 hypervariable regions. The molecular size and the purity of the DNA extracts were analyzed by agarose gel electrophoresis (1%). PCRs were carried out in 50μl reactions using 0.5–5 μl of DNA template, 2 mM MgCl_2_, 0.25 μM of each primer (forward and reverse), 0.25 mM dNTP and 1.25 U Taq polymerase, completed with ultrapure water. Negative controls containing ultrapure water instead of DNA template were performed at all PCR steps. Amplifications were confirmed by agarose gel electrophoresis run. DNA sequencing was carried out with the Illumina MiSeq by Research and Testing Laboratory (Lubbock, Texas).

### 2.5 Sequence data and diversity analysis

Paired-ends raw reads (2 x 250) were merged, quality-filtered and assigned to taxa after primers trimming, sequence clustering and chimera checking using the Mothur pipelines [42]. Clusters were assigned with the Silva 128 16S rRNA database [43] and clusters that did not belong to Bacteria kingdom were removed, as well as chloroplast and mitochondrial sequences. Operational Taxonomic Units (OTUs) were defined as clusters sharing 97% of sequence identity. The taxonomy assignments were completed using the SILVA v.128 database (https://www.arb-silva.de/documentation/release-128/). Bacterial sequences were randomly resampled in the OTU file to enable comparison between samples, by normalizing the number of sequences between samples to the sample with the fewest sequences (n = 468) using MacQiime 1.9.0 (single_rarefaction.py). All further analyses were performed on the randomly resampled OTU table.

OTUs richness was estimated by a non-parametric estimator of Chao1. The Jaccard dissimilarity matrix was used to visualize patterns in the community composition [44] by producing a Principal Coordinates Analysis (PcoA) plot among all samples [45]. Statistical analyses were done with the *vegan* package [46] in *R studio* 1.1.456 (R Development Core Team). The *ggplot2* package [47] was used in *R studio* to build boxplots with number of OTUs between MFs and seawater samples, as well as the PCoA plots.

## 3. Result and discussion

### 3.1 Morphological analysis of microfibers and their associated organisms

**Fig 2:**
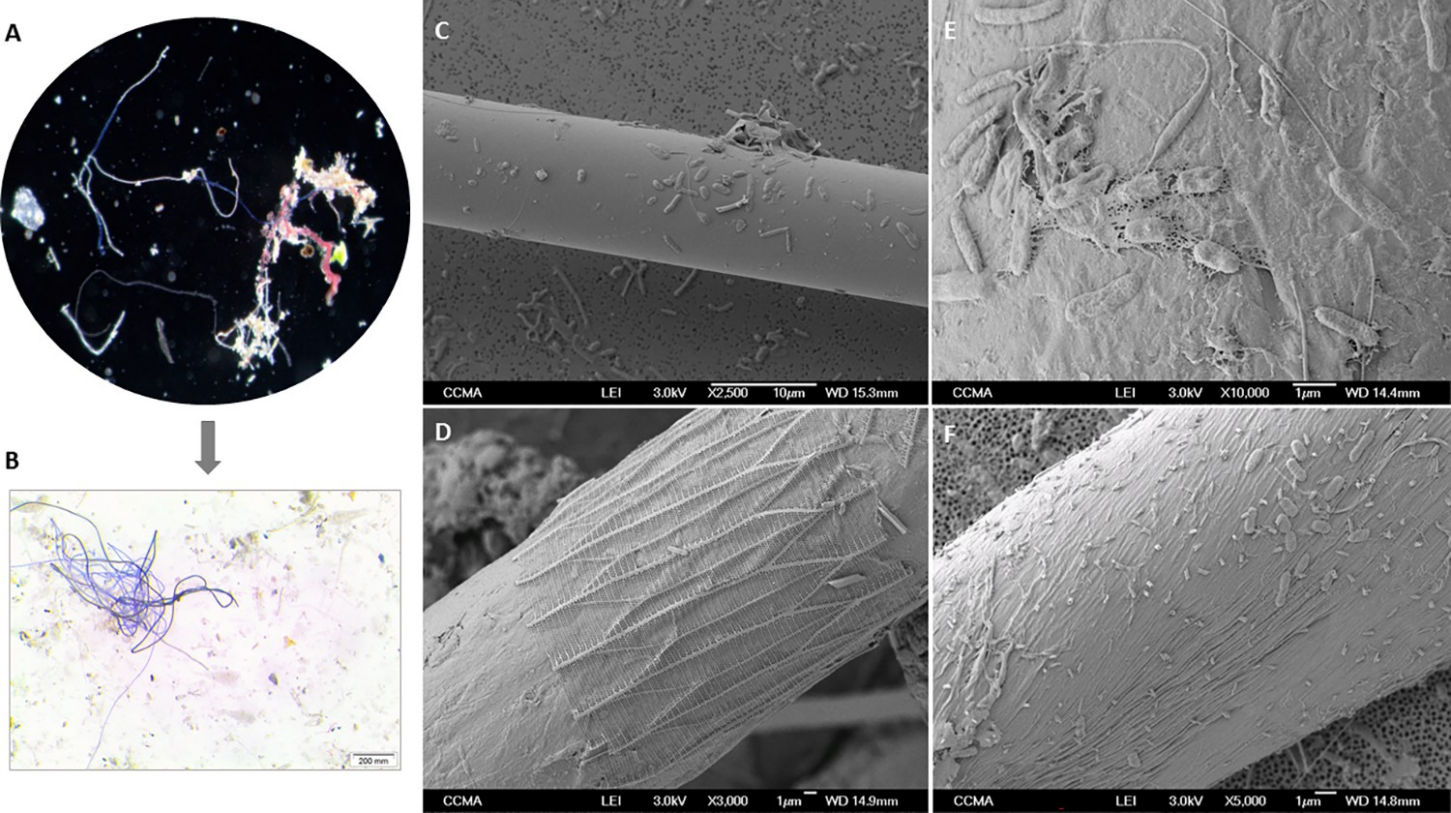
Optical microscopy of floating fibers sampled at the Mediterranean Sea (A, B), and Scanning Electron Microscopy (SEM) images of their attached bacteria, with elongated and rounded cells, as well as Extracellular Polymeric Substances (EPS) (C-F).

Optical microscopy of MFs showed a variety of MFs in all samples, in different sizes, thicknesses and colors, with an average length of 939 ± 1011 (size range from 52 to 6018 μm (Figs 2A and 2B, S1). SEM analysis showed that many MFs presented signs of degradation, including cracks and pitting. Bacterial cells were the most commonly associated organisms with the MFs. Although we also have observed many diatoms, it was not possible to estimate their abundance on MFs with the magnifications we used to capture the images, due the large size range of this group. Extracellular Polymeric Substances (EPS) were regularly observed (Fig 2C–2F). The average number of bacteria on the surface of a fiber was 8 ± 5.9 cells 100μm^-2^; extrapolating it to an entire fiber, this represents 2663 ± 1981 bacteria per fiber, revealing a strong bacterial presence on MFs. To date, only a few studies have quantified the surface area colonized by bacteria on microplastics in the marine environment [48, 49]. Our counts, although on different types of MFs and not only microplastics, yielded results in the same range as the above-mentioned studies in the Mediterranean, respectively 0.5 and 4.4 cells 100μm^-2^.

### 3.2 Description of microfibers-associated bacteria

**Fig 3:**
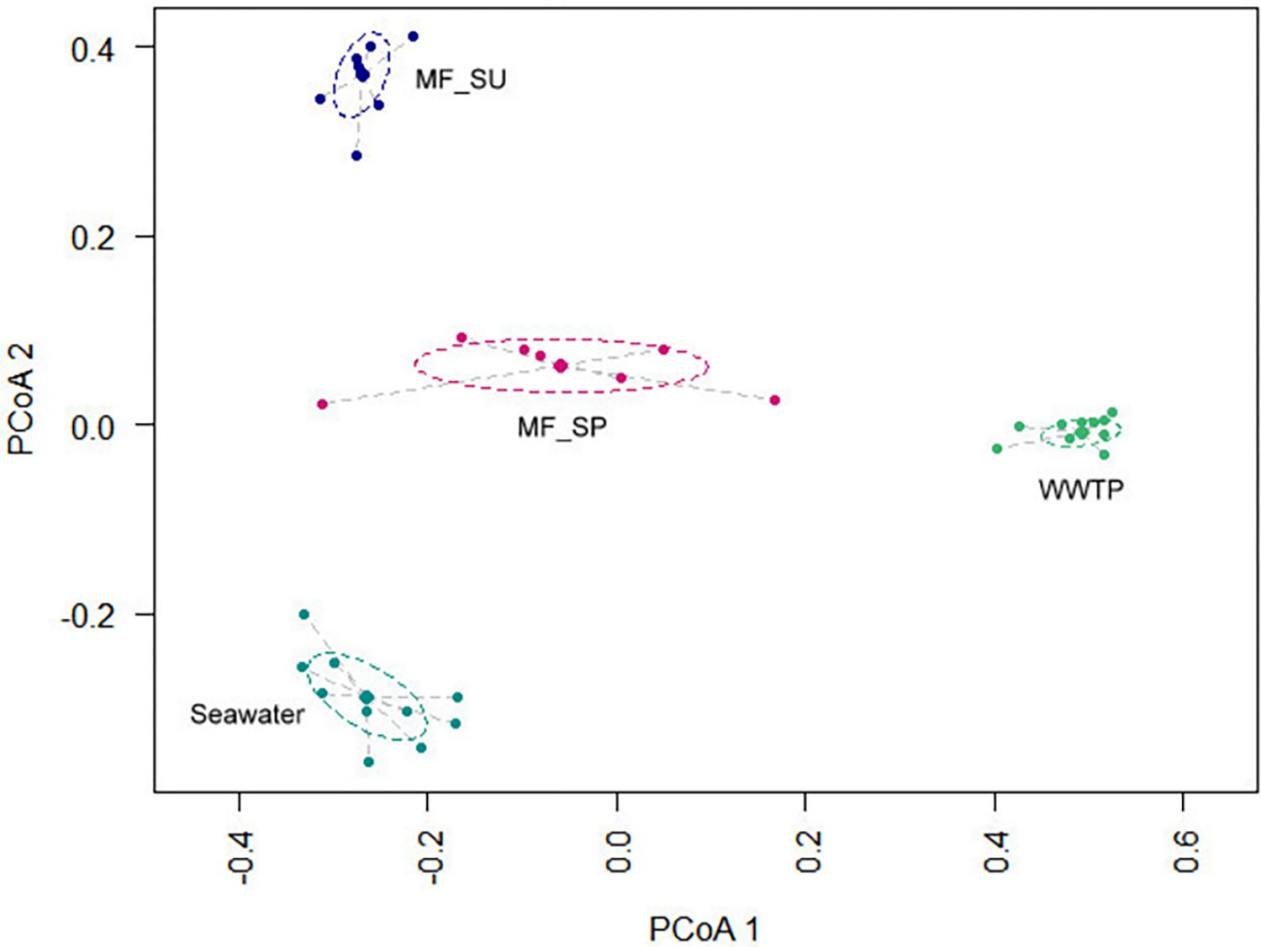
Principal coordinate analysis (PCoA) plot showing dissimilarities among the bacterial community composition attached to microfibers from summer (MF_SU) and spring (MF_SP) seasons, the free-living bacteria from the Seawater and from the Haliotis Waste Water Treatment Plant (WWTP). Samples were grouped based on Jaccard distance matrix.

We found a total of 195 bacterial OTUs belonging to twelve phyla and one unclassified bacterium. PCoA analysis showed clear distinctions between the bacterial community structure attached to MFs in comparison to seawater and the WWTP samples (Fig 3). WWTP samples were dominated by *Cloacibacterium normanense* (Bacteroidetes; Flavobacteriia) and *Arcobacter cryaerophilus* (Proteobacteria; Epsilonproteobacteria), both representing less than 0.01% of bacteria we found attached to MFs or seawater samples. Previous studies showed significant differences between bacterial assemblages on microplastics, borosilicate spheres, and bacterial communities in WWTP effluent water [50]. The presence of freshwater in WWTP, in opposition of the salt water found in coastal regions, could be the main factor distinguishing these populations. For that reason, we choose to exclude WWTP samples from further analysis as the description of WWTP bacteria was not the main goal of the present study.

**Fig 4:**
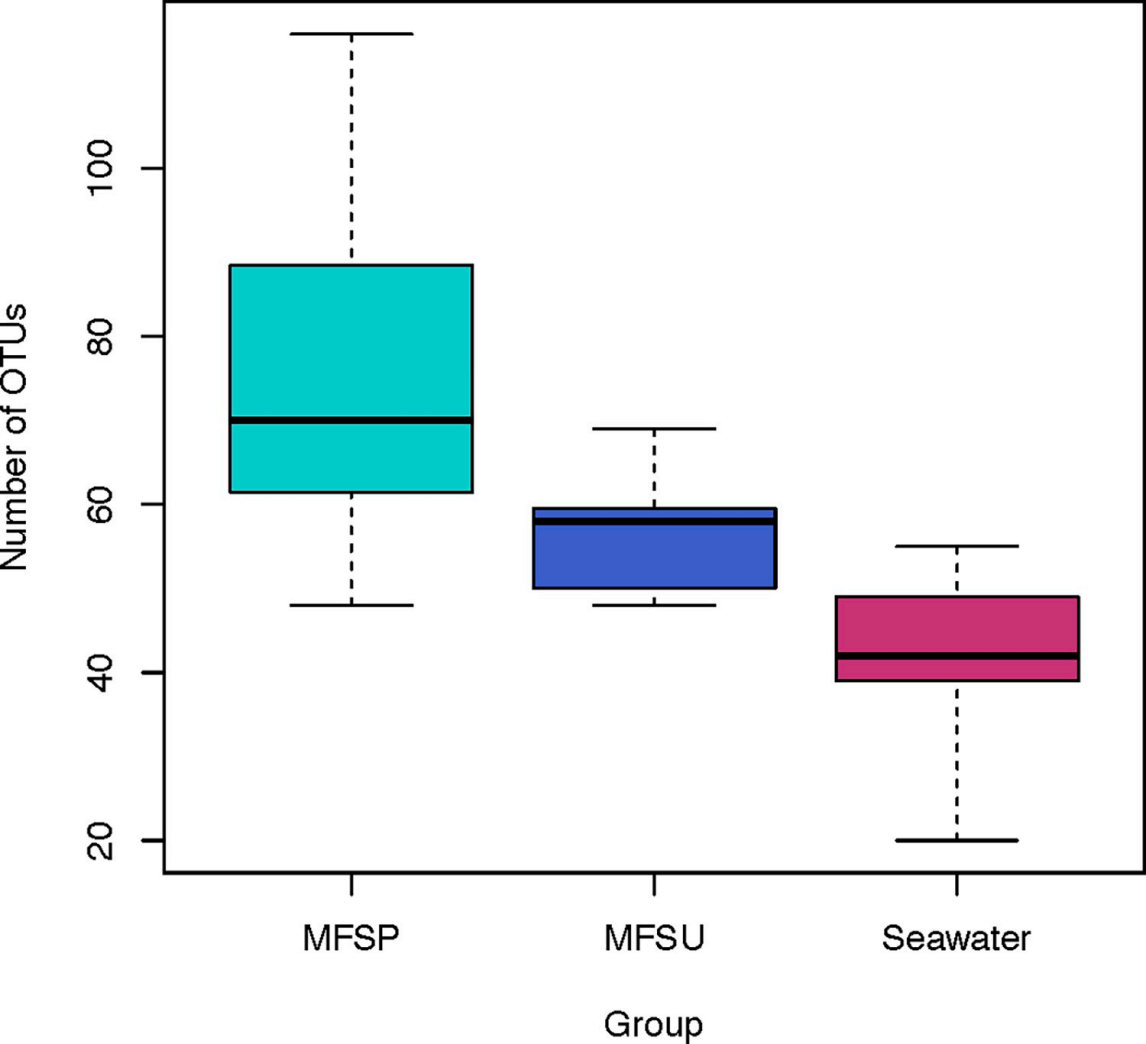
Number of observed OTUs per group of microfibers from spring (MFSP) and summer (MFSU) seasons, as well as from the Seawater, obtained from 16S amplicon sequence library, with significant difference between microfibers and seawater samples (Kruskal-Wallis, p < .01).

The OTUs richness among MFs and seawater samples was significantly different (Kruskal-Wallis, p < 0.01), being higher on MFs (Fig 4). Free-living bacterial community was dominated by (Alpha) Proteobacteria, Bacteroidetes, and unclassified Cyanobacteria, divided into eight OTUs that together represented 84% of free-living prokaryotes, all of them being frequent in all seawater samples. *Candidatus Pelagibacter* was the most abundant species (relative abundance of 28%). This is a ubiquitous bacteria member of the SAR11 clade, and indeed is one of the most abundant microbial plankton cells [51].

MFs-attached bacteria showed a different community composition, dominated by eight (Gamma or Alpha) Proteobacteria OTUs that represented 80% of the prokaryotes associated to MFs. *Alteromonas* sp. (Gammaproteobacteria) was the most abundant OTU (48% of reads), frequent in all MFs samples. This taxon is commonly found as the most abundant OTU in the marine plastisphere worldwide [45]. In addition, marine *Alteromonas spp* exhibit algicidal activity, which may attack cells and kill or lyse nearby microalgal cells [51]. In our study area, the putative pathogen *Vibrio parahaemolyticus* was the second most abundant OTU (7%) associated to MFs, with frequency of occurrence (FO) of 71% in MFs samples. Another *Vibrio* sp. was also highly frequent (FO 86%), even with low abundance (4.4%).

**Fig 5:**
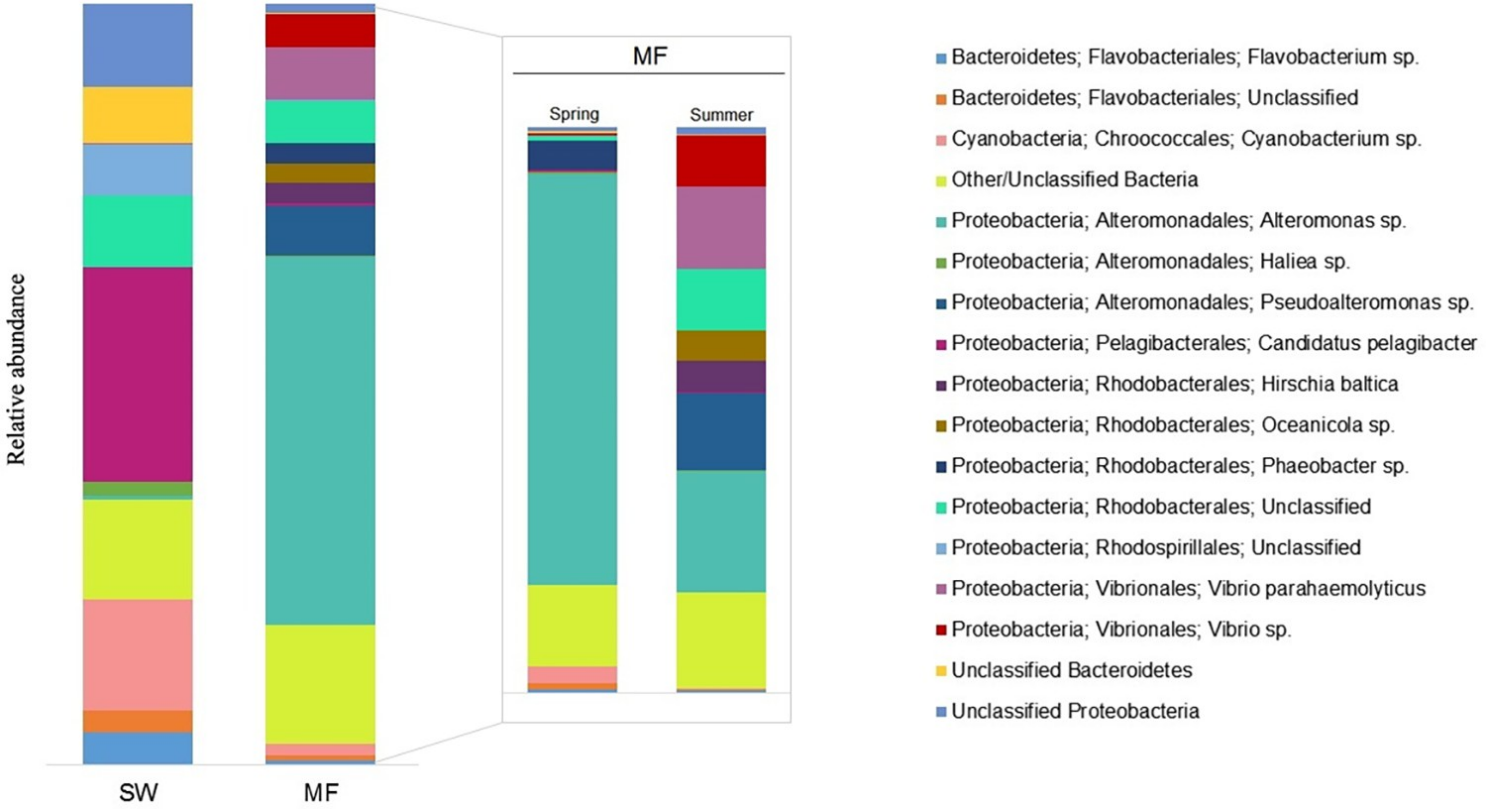
Bar chart showing taxonomic composition and dominance of a relatively small number of abundant OTUs, for seawater (SW) and floating microfibers (MF) in the Mediterranean Sea. Similarities between microfibers collected during spring and summer seasons are detailed. For more clarity, OTUs representing less than 1% or unclassified at the class level are grouped in the Other/Unknown series.

Comparison between summer and spring seasons (Fig 5) showed that four individual OTUs contributed to 68% of the dissimilarity among the MFs samples, with *Alteromonas* sp. dominating in the spring, while during the summer *Pseudoalteromonas* sp. and *Vibrio parahaemolyticus* were more representative. Considering the relevance of this taxon, we discuss the *Vibrio* spp. in a separate section hereafter. *Alteromonas* sp. was previously identified as an organic particle-attached group [52], whereas *Pseudoalteromonas* sp. is frequently associated to marine algae [53]. These associations support the fact that the plastisphere may be a self-sufficient ecosystem [54], with many ecological relations among its members that include symbionts, saprotrophs and parasites [55]. The communities we have identified living on MFs in the Mediterranean Sea form distinct groups in relation to the sampling seasons (summer and spring) (Fig 4). This is in accordance to what has been demonstrated in other regions [56–58] to plastisphere organisms, as they are highly dependent on environmental factors [28].

Other groups of bacteria found attached to MFs belong to the genera *Marinobacter* (FO 71%), *Pseudomonas* (FO 50%), *Acidovorax* and *Clostridium* (FO 43% each), *Acinetobacter* and *Comamonas* (FO 29% each), some of them described as able of biodegrading various types of polymers [59]. For example, a *Pseudomonas* strain has been shown to have enzymes such as monooxygenase that play a central role in polyethylene degradation [60]. The richness of OTUs estimated by Chao 1 shows that a very high diversity of bacteria exists on MFs, highlighting a vast and largely unknown functional potential. Although our data indicate the presence of these taxa associated to MFs in the Mediterranean, they do not allow us to assess the functionality of their genes. The identification of mechanisms involved on pathogenicity and biodegradation of synthetic polymers is therefore a priority issue [61].

Many bacteria found associated to MFs in the Mediterranean Sea were previously identified as potential animal and/or human pathogens, such as *Tenacibaculum*, *Vibrio* and *Pseudomonas* species [62]. These groups were also described as part of the marine plastisphere in the North/Baltic Seas [33, 63, 64], South Atlantic [65] and North Pacific [31] oceans. As mentioned above, the presence of *Vibrio* has been confirmed in various sampled fibers recovered in your sampling, which included both synthetic and natural fibers. Although our results are not exclusively related to synthetic fibers, they do reinforce the role of plastics, including synthetic microfibers, in harboring potentially harmful organisms.

### 3.3 Vibrio spp. associated to microfibers in the Mediterranean Sea

To date, only a few studies have reported bacteria that could be a putative human pathogen on MFs or other types of microplastics in Mediterranean waters [49, 54]. This study is the first to describe V. *parahaemolyticus* on floating MFs at the region. Members of the genus *Vibrio* were also found on plastic debris at a Mediterranean beach (Calvi, Corsica) with the pathogenic *V*. *splendidus* accounting for up to 70% of the total reads [54]. Other studies detected *V*. *parahaemolyticus* attached to microplastics, one in the South Atlantic Ocean [65], and another in the North Sea/Baltic Sea [35], including polyethylene fibers, polyethylene films and polypropylene fragments. Kirstein and colleagues [35] also identified this species in the surrounding water where microplastics were sampled, thus suggesting that the seawater could be a potential reservoir of *Vibrio* species. Recently, Kesy et al. [64] showed that the colonization of plastics by *Vibrio* sp. was observed within the first hour of exposure of these materials *in situ*, highlighting that *Vibrio* sp. are amongst the very first colonizers on plastics.

In our survey, this putative pathogen represented around 6% of the total community in the MFs fraction, mostly present during the summer, and it counted for 28% of reads in a single sample from a coastal station; this taxon was not found in the seawater samples from the offshore Dyfamed station, and it was found in a very low abundance attached to the MFs (0.01%) from Dyfamed, emphasizing that the risk of contamination may be higher in areas of high anthropogenic impact. In the Baltic Sea, a positive correlation was found between *Vibrio* abundances and the presence of cities with more than 100,000 inhabitants [64]. *Vibrio parahaemolyticus* can potentially infect humans when ingesting raw or partially cooked shellfish [66] This is noteworthy since *Vibrio* spp are rarely found in concentrations that can account for more than 1% of the community attached to plastics [67].

Other potentially pathogenic *Vibrio* species not related to human diseases were observed on microplastics in the Mediterranean Sea, such as *V*. *anguillarum*, *V*. *harveyi*, *V*. *pectinicida*, *V*. *xiamenensis* but again as a low percentage of the community (**<** 0.1%) [48]. In our samples, *Enterovibrio calviensis* (first described in the Mediterranean as *Vibrio* sp.) was frequent in all MFs samples, in low abundances, but this is a species resistant to antibiotics (lincomycin, oxacillin and spectinomycin), and a facultative anaerobe, able to reduce nitrate to nitrite [68]. Vibrionales are generally known to express antagonistic activities, being the most prolific producers of inhibiting materials but also the most resistant to them [69]. In addition, some *Vibrio* species are capable of degrading toxic polycyclic aromatic hydrocarbons (PAHs] in polluted marine sediments [70]. The evaluation of the potential virulence of environmental strains of *Vibrio parahaemolyticus* has shown that they have the ability to regulate newly acquired virulence factors, e.g. in response to the temperature, but the ability to adapt to a human host environment has not yet been demonstrated [71]. Although abundant in the Mediterranean Sea and occurring in many areas in the Baltic Sea, metaproteomic analyses have shown that, in the Vibrionaceae family, proteins related to virulence processes are not very active [54, 72].

Furthermore, while it is now established that there is a specific microbiome growing on plastics that differs from the seawater (free-living and associated with organic particles), there is less evidence regarding the differences between natural and synthetic substrates [67]. A study of three marine ecosystems under various environmental conditions found that a large proportion of the OTUs present on plastics were absent in non-plastic particles and in the seawater [45]. Regarding *Vibrio* sp, they are known to be associated with various natural substrates such as wood, cellulose or glass, and their abundance appears to be low on plastic debris compared to natural ones [31, 67]. Nevertheless, in our study, we were able to confirm the presence of *Vibrio* in the MFs, although it cannot be established directly on synthetic or natural fibers. A recent meta-analysis of several environments shows that the variety of potentially pathogenic species found on microplastics is comparable to natural particles [37]. Many questions remain open as to whether, rather than a selection of distinct microbial colonizers, persistent plastic debris could lead to sustain selection of biofilms, thereby increasing the risk of pathogen transport and disease occurrence [37].

### 3.4 Impacts of microfibers and their attached bacteria in the Mediterranean Sea

In a global fiber study covering surface waters of six ocean basins, the Mediterranean Sea had the highest concentrations (median of 4.2 fibers L^-1^) [18]. The median concentration of synthetic microfibers in the western basin was even higher (10.7 fibers L^-1^), as the characterization by ATR-FT-IR revealed that they represent 14–50% of the raw materials [19]. This high concentration of fibers could be related to the higher density of Mediterranean waters (generally >1.026 g cm^-3^), which allows fibers such as polyamides, commonly used in the textile industry and fishing (density between 1.02 and 1.15 g cm^-3^), to remain in the upper layers in higher proportions [15], when compared to other seas [73, 74]. Another explanation is related to their shape, as vertical advection velocities are lower for fibrous microplastics than for sheets and other shapes [75], which may contribute to a longer residence time for these fibrous materials, favoring their transport as potential vectors for organisms, including putative pathogens.

Studies have shown that temperature and salinity affect *Vibrio* species, in particular temperature has a significant correlation with the increase of *Vibrio* spp. [33, 76, 77]. In the Baltic Sea, gradual warming of the water favored the appearance of potentially pathogenic *Vibrio* and the emergence of infections [78]. Higher temperature was also positively correlated with the density of *V*. *parahaemolyticus* in oysters [66]. A recent study showed that the increase of seawater temperature has an important influence on the adhesion properties of free-living *V*. *parahaemolyticus* to plastic, with all analyzed factors being transiently expressed in 27°C and even more upregulated at 31°C, emphasizing the role of climate change in the spread of this pathogenic bacteria [79]. In the Thau Lagoon (Gulf of Lyon), episodes of massive oyster mortality coinciding with single or double infections involving mainly OsHV-I and *Vibrio splendidus* have been observed when seawater temperature is above 24°C [80].

Indeed, the temperature during the summer cruise ranged from 25.2 to 26.5°C. During this season, although low runoff may decrease plastic transport from freshwater systems [81], the population double in this area as this is the touristic season. The western coasts of the Mediterranean are among the most densely populated areas, with population size expected to increase nearly twofold over the next decade [38]. Much of the world’s plastic waste enters the oceans at heavily populated coastal sites and near wastewater drainage systems. While the increased spread of potentially pathogenic bacteria on floating plastics is a real threat to low-income countries [82], the presence of putative pathogenic *Vibrio* in a highly persistent (or ubiquitous) anthropogenic particle (that can be transported long distances) in the Mediterranean Sea is also becoming a major environmental concern.

## 4. Final remarks

Our study shows clear distinctions between bacterial communities on MFs compared to the free-living bacteria inhabiting surface waters in the Mediterranean Sea. It highlights a strong colonization of MFs by microorganisms and reports the first occurrence of the pathogenic *Vibrio parahaemolyticus* attached to floating MFs, including synthetic ones, in surface waters of Northwestern Mediterranean Sea, especially during the summer. We have shown that microfibers, both natural and synthetic, have the ability to host bacterial species, including potential pathogens, although the comparison is not sufficient to draw conclusions on the enrichment of certain bacterial species on microplastics.

This study raises the question of whether the increasing amount of persistent plastic waste in the environment may influence the dynamics of various hitchhikers offering greater transport opportunities, thus leading to an increased risk of contamination compared to other short-lived natural particles, such as wood or sediments. Considering that synthetic fibers can serve as a vector for potentially pathogenic microorganisms and other pollutants in the ocean, due to their longevity, this type of pollution may have ecological and economic consequences. These results on the characteristics of microbial assemblages are valuable for future assessments of the health risks associated with plastic pollution, as their presence may pose a threat to swimming and seafood consumption.

The Mediterranean is under constant anthropogenic pressure regarding pollution, as well as the consequences of climate change exceeding global trends for most variables, with waters warming faster than the rest of the ocean, especially during the summer months [83]. These changing conditions can lead to shifts in marine microbial community structure, including particle colonizers in areas with high levels of plastic pollution. Further studies are needed to target genes associated with virulence to prevent the spread of diseases. Human discharges of chemicals and plastics into continents and oceans have reached a critical threshold, and plastic pollution meets the criteria for planetary boundary threats [84].

## Supporting information

S1 File (DOCX)

## References

[1] Plastics Europe GMR, Conversio Market & Strategy GmbH. Plastics—the Facts 2019. 2019;14, 35. Available from: https://www.plasticseurope.org/en/resources/market-data

[2] Geyer R. Production, use, and fate of synthetic polymers. Plastic Waste and Recycling. Elsevier Inc.; 2020. 13–32 p. Available from: 10.1016/B978-0-12-817880-5.00002-5

[3] Rochman CM. Microplastics research—from sink to source in freshwater systems. Science. 2018;360(6384):28–9. 29622640 10.1126/science.aar7734

[4] Turner A, Arnold R, Williams T. Weathering and persistence of plastic in the marine environment: Lessons from LEGO. Environ Pollut. 2020;262:114299. Available from: doi: 10.1016/j.envpol.2020.114299 32163808

[5] Thompson RC, Olsen Y, Mitchell RP, Davis A, Rowland SJ, John AWG, et al. Lost at Sea: Where Is All the Plastic? Science. 2004;304(3). doi: 10.1126/science.1094559 15131299

[6] van Sebille E, Wilcox C, Lebreton L, Maximenko N, Hardesty BD, van Franeker JA, et al. A global inventory of small floating plastic debris. Environ Res Lett. 2015;10(12):124006. Available from: http://stacks.iop.org/1748-9326/10/i=12/a=124006?key=crossref.56648155cc7e4c5554d3217914246a05

[7] Jambeck JR, Geyer R, Wilcox C, Siegler TR, Perryman M, Andrady A, et al. Plastic waste inputs from land into the ocean. Science. 2015;347(6223):768–71. 25678662 10.1126/science.1260352

[8] Isobe A, Azuma T, Cordova MR, Cózar A, Galgani F, Hagita R, et al. A multilevel dataset of microplastic abundance in the world’s upper ocean and the Laurentian Great Lakes. Microplastics and Nanoplastics. 2021;1:1–14.

[9] Borrelle SB, Ringma J, Law KL, Monnahan CC, Lebreton L, McGivern A, et al. Mitigate Plastic Pollution. Science. 2020;:1515–8. Available from: http://science.sciencemag.org/content/369/6510/1515 32943526 10.1126/science.aba3656

[10] The Fiber Year 2019. 2018;(13):2018–20.

[11] Axelsson C, van Sebille E. Prevention through policy: Urban macroplastic leakages to the marine environment during extreme rainfall events. Mar Pollut Bull. 2017;124(1):211–27. Available from: doi: 10.1016/j.marpolbul.2017.07.024 28755809 PMC5667635

[12] Cesa FS, Turra A, Baruque-Ramos J. Synthetic fibers as microplastics in the marine environment: A review from textile perspective with a focus on domestic washings. Sci Total Environ. 2017;598:1116–29. Available from: doi: 10.1016/j.scitotenv.2017.04.172 28482459

[13] Dris R, Gasperi J, Saad M, Mirande C, Tassin B. Synthetic fibers in atmospheric fallout: A source of microplastics in the environment? Mar Pollut Bull. 2016;104(1–2):290–3. Available from: doi: 10.1016/j.marpolbul.2016.01.006 26787549

[14] Gasperi J, Wright SL, Dris R, Collard F, Mandin C, Guerrouache M, et al. Microplastics in air: Are we breathing it in? Curr Opin Environ Sci Heal. 2018;1:1–5. Available from: 10.1016/j.coesh.2017.10.002

[15] Trainic M, Flores JM, Pinkas I, Pedrotti ML, Lombard F, Bourdin G, et al. Airborne microplastic particles detected in the remote marine atmosphere. Commun Earth Environ. 2020;1(1):1–10. Available from: 10.1038/s43247-020-00061-y

[16] Cole M, Lindeque P, Halsband C, Galloway TS. Microplastics as contaminants in the marine environment: A review. Mar Pollut Bull. 2011;62(12):2588–97. doi: 10.1016/j.marpolbul.2011.09.025 22001295

[17] Carr SA. Sources and dispersive modes of micro-fibers in the environment. Integr Environ Assess Manag. 2017;13(3):466–9. doi: 10.1002/ieam.1916 28440926

[18] Suaria G, Achtypi A, Perold V, Lee JR, Pierucci A, Bornman TG, et al. Microfibers in oceanic surface waters: A global characterization. Sci Adv. 2020;6(23). doi: 10.1126/sciadv.aay8493 32548254 PMC7274779

[19] Pedrotti ML, Petit S, Eyheraguibel B, Kerros ME, Elineau A, Ghiglione JF, et al. Pollution by anthropogenic microfibers in North-West Mediterranean Sea and efficiency of microfiber removal by a wastewater treatment plant. Sci Total Environ. 2021;758. doi: 10.1016/j.scitotenv.2020.144195 33338794

[20] Henry B, Laitala K, Klepp IG. Microfibres from apparel and home textiles: Prospects for including microplastics in environmental sustainability assessment. Sci Total Environ. 2019;652:483–94. Available from: doi: 10.1016/j.scitotenv.2018.10.166 30368178

[21] Zambrano MC, Pawlak JJ, Richard A. Venditti. Effects of Chemical and Morphological Structure on Biodegradability of Fibers, Fabrics, and Other Polymeric Materials. BioResources. 2020; 9786–833.

[22] Mishra S., Singh R. P., Rath C. C., & Das A. P. (2020). Synthetic microfibers: Source, transport and their remediation. Journal of Water Process Engineering, 38, 101612.

[23] Barrows APW, Cathey SE, Petersen CW. Marine environment microfiber contamination: Global patterns and the diversity of microparticle origins. Environ Pollut. 2018;237:275–84. Available from: doi: 10.1016/j.envpol.2018.02.062 29494921

[24] Rebelein A, Int-Veen I, Kammann U, Scharsack JP. Microplastic fibers—Underestimated threat to aquatic organisms? Sci Total Environ. 2021;777:146045. Available from: doi: 10.1016/j.scitotenv.2021.146045 33684771

[25] Teuten EL, Saquing JM, Knappe DRU, Barlaz MA, Jonsson S, Bjorn A, et al. Transport and release of chemicals from plastics to the environment and to wildlife. Philos Trans R Soc B Biol Sci. 2009;364:2027–2045 10.1098/rstb.2008.0284 PMC2873017 19528054

[26] Baini M, Martellini T, Cincinelli A, Campani T, Minutoli R, Panti C, et al. First detection of seven phthalate esters (PAEs) as plastic tracers in superficial neustonic/planktonic samples and cetacean blubber. Anal Methods. Royal Society of Chemistry. 2017;9(9):1512–1520.

[27] Landrigan PJ, Stegeman JJ, Fleming LE, Allemand D, Anderson DM, Backer LC, et al. Human health and ocean pollution. Ann Glob Heal. 2020;86(1):1–64. doi: 10.5334/aogh.2831 33354517 PMC7731724

[28] Amaral-Zettler LA, Zettler ER, Mincer TJ. Ecology of the plastisphere. Nat Rev Microbiol. 2020; Available from: 10.1038/s41579-019-0308-0 31937947

[29] Zettler ER, Mincer TJ, Amaral-Zettler LA. Life in the “Plastisphere”: microbial communities on plastic marine debris. Environ Sci Technol. 2013;47:7137. doi: 10.1021/es401288x 23745679

[30] Amaral-Zettler LA, Zettler ER, Slikas B, Boyd GD, Melvin DW, Morrall CE, et al. The biogeography of the Plastisphere: implications for policy. Front Ecol Environ. 2015;13(10):541–6. Available from: http://www.esajournals.org/doi/abs/10.1890/150017

[31] Bryant JA, Clemente TM, Viviani DA, Fong AA, Thomas KA, Kemp P, et al. Diversity and Activity of Communities Inhabiting Plastic Debris in the North Pacific Gyre. mSystems. 2016;1(3):e00024–16. Available from: http://msystems.asm.org/lookup/doi/10.1128/mSystems.00024-16 27822538 10.1128/mSystems.00024-16 PMC5069773

[32] Dussud C, Hudec C, George M, Fabre P, Higgs P, Bruzaud S, et al. Colonization of non-biodegradable and biodegradable plastics by marine microorganisms. Front Microbiol. 2018; 9:1571. Available from: doi: 10.3389/fmicb.2018.01571 30072962 PMC6058052

[33] Oberbeckmann S, Kreikemeyer B, Labrenz M, Harrison JP. Environmental Factors Support the Formation of Specific Bacterial Assemblages on Microplastics. 2018;8:1–12. 10.3389/fmicb.2017.02709 PMC5785724 29403454

[34] Keswani A, Oliver DM, Gutierrez T, Quilliam RS. Microbial hitchhikers on marine plastic debris: Human exposure risks at bathing waters and beach environments. Mar Environ Res. 2016;118:10–9. Available from: doi: 10.1016/j.marenvres.2016.04.006 27128352

[35] Kirstein I V., Kirmizi S, Wichels A, Garin-Fernandez A, Erler R, Löder M, et al. Dangerous hitchhikers? Evidence for potentially pathogenic *Vibrio* spp. on microplastic particles. Mar Environ Res. 2016;120:1–8. Available from: 10.1016/j.marenvres.2016.07.004 27411093

[36] Smith M, Love DC, Rochman CM, Neff RA. Microplastics in Seafood and the Implications for Human Health. Curr Environ Heal reports. Current Environmental Health Reports. 2018;5(3):375–86. doi: 10.1007/s40572-018-0206-z 30116998 PMC6132564

[37] Bowley J, Baker-Austin C, Porter A, Hartnell R, Lewis C. Oceanic Hitchhikers–Assessing Pathogen Risks from Marine Microplastic. Trends Microbiol. 2021;29(2):107–16. Available from: doi: 10.1016/j.tim.2020.06.011 32800610

[38] UN/MAP. Mediterranean Quality Status Report. Mediterr Action Plan Barcelona Conv. 2017;539. Available from: https://www.medqsr.org/sites/default/files/inline-files/2017MedQSR_Online_0.pdf

[39] Bianchi CN, Morri C. Marine biodiversity of the Mediterranean Sea: Situation, problems and prospects for future research. Mar Pollut Bull. 2000;40(5):367–76.

[40] Deudero S, Alomar C. Mediterranean marine biodiversity under threat: Reviewing influence of marine litter on species. Mar Pollut Bull. 2015;98(1–2):58–68. Available from: doi: 10.1016/j.marpolbul.2015.07.012 26183308

[41] Hansson L, Agis M, Maier C, Weinbauer MG. Community composition of bacteria associated with cold-water coral *Madrepora oculata*: Within and between colony variability. Mar Ecol Prog Ser. 2009;397:89–102.

[42] Schloss PD, Westcott SL, Ryabin T, Hall JR, Hartmann M, Hollister EB, et al. Introducing mothur: open-source, platform-independent, community-supported software for describing and comparing microbial communities. Appl Environ Microbiol. 2009;75(23):7537–41. doi: 10.1128/AEM.01541-09 19801464 PMC2786419

[43] Quast C, Pruesse E, Yilmaz P, Gerken J, Schweer T, Yarza P, et al. The SILVA ribosomal RNA gene database project: Improved data processing and web-based tools. Nucleic Acids Res. 2012;41. doi: 10.1093/nar/gks1219 23193283 PMC3531112

[44] Kirstein I V., Wichels A, Krohne G, Gerdts G. Mature biofilm communities on synthetic polymers in seawater—Specific or general? Mar Environ Res. 2018;142:147–54. Available from: 10.1016/j.marenvres.2018.09.028 30337052

[45] Scales BS, Cable RN, Duhaime MB, Gerdts G, Fischer F, Fischer D, et al. Cross-Hemisphere Study Reveals Geographically Ubiquitous, Unexplored Biosphere. Am Soc Microbiol. 2021;6(3). 10.1128/mSphere.00851-20 PMC8265672 34106771

[46] Oksanen J, Blanchet FG, Friendly M, Kindt R, Legendre P, Mcglinn D, et al. Package “vegan” Title Community Ecology Package. Community Ecol Packag. 2019;2(9). Available from: https://cran.r-project.org/web/packages/vegan/vegan.pdf

[47] Wickham H. ggplot2 by Hadley Wickham. Media [Internet]. 2009;35:211. Available from: http://had.co.nz/ggplot2/book.

[48] Dussud C, Meistertzheim AL, Conan P, Pujo-Pay M, George M, Fabre P, et al. Evidence of niche partitioning among bacteria living on plastics, organic particles and surrounding seawaters. Environ Pollut. 2018;236:807–16. Available from: doi: 10.1016/j.envpol.2017.12.027 29459335

[49] Amaral-Zettler LA, Ballerini T, Zettler ER, Asbun AA, Adame A, Casotti R, et al. Diversity and predicted inter- and intra-domain interactions in the Mediterranean Plastisphere. Environ Pollut. 2021;286:117439. doi: 10.1016/j.envpol.2021.117439 34438479

[50] Martínez-Campos S, González-Pleiter M, Fernández-Piñas F, Rosal R, Leganés F. Early and differential bacterial colonization on microplastics deployed into the effluents of wastewater treatment plants. Sci Total Environ. 2021;757:143832. Available from: doi: 10.1016/j.scitotenv.2020.143832 33246729

[51] Morris RM, Rappé MS, Connon SA, Vergin KL, Siebold WA, Carlson CA, et al. SAR11 clade dominates ocean surface bacterioplankton communities. Nature. 2002;420(6917):806–10. doi: 10.1038/nature01240 12490947

[52] Cho JY. Algicidal activity of Marine *Alteromonas* sp. KNS-16 and isolation of active compounds. Biosci Biotechnol Biochem. 2012;76(8):1452–8. 22878186 10.1271/bbb.120102

[53] Lin X, Yang B, Shen J, Du N. Biodegradation of crude oil by an arctic psychrotrophic bacterium *Pseudoalteromomas* sp. P29. Curr Microbiol. 2009;59(3):341–5. 19543945 10.1007/s00284-009-9440-9

[54] Delacuvellerie A, Géron A, Gobert S, Wattiez R. New insights into the functioning and structure of the PE and PP plastispheres from the Mediterranean Sea. Environ Pollut. 2021;118678. Available from: doi: 10.1016/j.envpol.2021.118678 34915097

[55] Lacerda ALdF Proietti MC, Secchi ER Taylor JD. Diverse groups of fungi are associated with plastics in the surface waters of the Western South Atlantic and the Antarctic Peninsula. Mol Ecol. 2020;29(10):1903–18. doi: 10.1111/mec.15444 32270556

[56] Oberbeckmann S, Loeder MGJ, Gerdts G, Osborn MA. Spatial and seasonal variation in diversity and structure of microbial biofilms on marine plastics in Northern European waters. FEMS Microbiol Ecol. 2014;90(2):478–92. doi: 10.1111/1574-6941.12409 25109340

[57] Oberbeckmann S, Osborn AM, Duhaime MB. Microbes on a bottle: Substrate, season and geography influence community composition of microbes colonizing marine plastic debris. PLoS One. 2016;11(8). doi: 10.1371/journal.pone.0159289 27487037 PMC4972250

[58] Zhang B, Yang X, Liu L, Chen L, Teng J, Zhu X, et al. Spatial and seasonal variations in biofilm formation on microplastics in coastal waters. Sci Total Environ. 2021;770:145303. Available from: doi: 10.1016/j.scitotenv.2021.145303 33515883

[59] Jacquin J, Cheng J, Odobel C, Pandin C, Conan P, Pujo-Pay M, et al. Microbial ecotoxicology of marine plastic debris: A review on colonization and biodegradation by the “plastisphere”. Front Microbiol. 2019;10:1–16. 31073297 10.3389/fmicb.2019.00865 PMC6497127

[60] Gyung Yoon M, Jeong Jeon H, Nam Kim M. Biodegradation of Polyethylene by a Soil Bacterium and AlkB Cloned Recombinant Cell. J Bioremediation Biodegrad. 2012;03(04).

[61] Wright RJ, Bosch R, Langille MGI, Christie-oleza JA. A Multi-OMIC Characterisation of Biodegradation and Microbial Community Succession Within the PET Plastisphere. Limnol Oceanogr Methods. 2020;2:1–25. Available from: http://doi.wiley.com/10.4319/lom.2004.2.365

[62] Hou D, Hong M, Wang Y, Dong P, Cheng H, Yan H, et al. Assessing the Risks of Potential Bacterial Pathogens Attaching to Different Microplastics during the Summer–Autumn Period in a Mariculture Cage. Microorganisms. 2021;9(9):1909. doi: 10.3390/microorganisms9091909 34576804 PMC8469625

[63] De Tender C, Schlundt C, Devriese LI, Mincer TJ, Zettler ER, Amaral-Zettler LA. A review of microscopy and comparative molecular-based methods to characterize “Plastisphere” communities. Anal Methods. Royal Society of Chemistry. 2017;9(14):2132–43. Available from: http://xlink.rsc.org/?DOI=C7AY00260B

[64] Kesy K, Labrenz M, Scales BS, Kreikemeyer B, Oberbeckmann S. Vibrio colonization is highly dynamic in early microplastic-associated biofilms as well as on field-collected microplastics. Microorganisms. 2021;9(1):1–13. 10.3390/microorganisms9010076 PMC7823642 33396691

[65] Lacerda ALdF, Taylor JD, Rodrigues L d. S, Kessler F, Secchi E, Proietti MC. Floating plastics and their associated biota in the Western South Atlantic. Sci Total Environ. 2022;805:150186. Available from: 10.1016/j.scitotenv.2021.150186 34818771

[66] Sobrinho PDSC, Destro MT, Franco BDGM, Landgraf M. Correlation between environmental factors and prevalence of *Vibrio parahaemolyticus* in oysters harvested in the southern coastal area of Sao Paulo state, Brazil. Appl Environ Microbiol. 2010;76(4):1290–3 20023076 10.1128/AEM.00861-09 PMC2820972

[67] Oberbeckmann S, Labrenz M. Marine Microbial Assemblages on Microplastics: Diversity, Adaptation, and Role in Degradation. Ann Rev Mar Sci. 2020;12:209–32. doi: 10.1146/annurev-marine-010419-010633 31226027

[68] Denner EBM, Vybiral D, Fischer UR, Velimirov B, Busse HJ. *Vibrio calviensis* sp. nov., a halophilic, facultatively oligotrophic 0.2 μm-filterable marine bacterium. Int J Syst Evol Microbiol. 2002;52(2):549–53. 11931167 10.1099/00207713-52-2-549

[69] Long RA, Azam F. Microscale patchiness of bacterioplankton assemblage richness in seawater. Aquat Microb Ecol. 2001;26(2):103–13.

[70] Hedlund BP, Staley JT. *Vibrio cyclotrophicus* sp. nov., a polycyclic aromatic hydrocarbon (PAH)-degrading marine bacterium. Int J Syst Evol Microbiol. 2001;51(1):61–6. 11211274 10.1099/00207713-51-1-61

[71] Mahoney JC, Gerding MJ, Jones SH, Whistler CA. Comparison of the pathogenic potentials of environmental and clinical *Vibrio parahaemolyticus* strains indicates a role for temperature regulation in virulence. Appl Environ Microbiol. 2010;76(22):7459–65. 20889774 10.1128/AEM.01450-10 PMC2976215

[72] Oberbeckmann S, Bartosik D, Huang S, Werner J, Hirschfeld C, Wibberg D, et al. Genomic and proteomic profiles of biofilms on microplastics are decoupled from artificial surface properties. Environ Microbiol. 2021;23(6):3099–3115. doi: 10.1111/1462-2920.15531 33876529

[73] Hidalgo-ruz V, Gutow L, Thompson RC, Thiel M. Microplastics in the Marine Environment: A Review of the Methods Used for Identification and Quantification. Environ Sci Technol. 2012;46:3060−3075. doi: 10.1021/es2031505 22321064

[74] Reisser J, Shaw J, Wilcox C, Hardesty BD, Proietti M, Thums M, et al. Marine Plastic Pollution in Waters around Australia: Characteristics, Concentrations, and Pathways. PLoS One. 2013;8(11):e80466. doi: 10.1371/journal.pone.0080466 24312224 PMC3842337

[75] Ballent A, Purser A, Mendes PDJ, Thomsen L. Physical transport properties of marine microplastic pollution. Biogeosciences Discuss. 2012;9(12):18755–98.

[76] Oberbeckmann S, Wichels A, Wiltshire KH, Gerdts G. Occurrence of *Vibrio parahaemolyticus* and *Vibrio alginolyticus* in the German Bight over a seasonal cycle. Int J Gen Mol Microbiol. 2011;100(2):291–307. 10.1007/s10482-011-9586-x 21598011

[77] Takemura AF, Chien DM, Polz MF. Associations and dynamics of vibrionaceae in the environment, from the genus to the population level. Front Microbiol. 2014;5:1–26. 24575082 10.3389/fmicb.2014.00038 PMC3920100

[78] Baker-Austin C, Trinanes JA, Taylor NGH, Hartnell R, Siitonen A, Martinez-Urtaza J. Emerging *Vibrio* risk at high latitudes in response to ocean warming. Nat Clim Chang. 2013;3(1):73–7. Available from: 10.1038/nclimate1628

[79] Billaud M, Seneca F, Tambutté E, Czerucka D. An Increase of Seawater Temperature Upregulates the Expression of *Vibrio parahaemolyticus* Virulence Factors Implicated in Adhesion and Biofilm Formation. Front Microbiol. 2022;13:1–10. 10.3389/fmicb.2022.840628 PMC8957992 35350627

[80] Cochennec-Laureau N, Baud J-P. Assessment of excess mortality in Pacific oysters (*Crassostrea gigas*) since 2008. Bull épidémiologique, santé Anim Aliment. 2008;42:2–5.

[81] Lebreton LCM, van der Zwet J, Damsteeg J-W, Slat B, Andrady A, Reisser J. River plastic emissions to the world’s oceans. Nat Commun. 2017;8:15611. Available from: http://www.nature.com/doifinder/10.1038/ncomms15611 28589961 10.1038/ncomms15611 PMC5467230

[82] Bastaraud A, Cecchi P, Handschumacher P, Altmann M, Jambou R. Urbanization and waterborne pathogen emergence in low-income countries: Where and how to conduct surveys? Int J Environ Res Public Health. 2020;17(2). doi: 10.3390/ijerph17020480 31940838 PMC7013806

[83] Cramer W, Guiot J, Fader M, Garrabou J, Gattuso JP, Iglesias A, et al. Climate change and interconnected risks to sustainable development in the Mediterranean. Nat Clim Chang. 2018;8(11):972–80.

[84] Persson L, Carney Almroth BM, Collins CD, Cornell S, de Wit CA, Diamond ML, et al. Outside the Safe Operating Space of the Planetary Boundary for Novel Entities. Environ Sci Technol. 2022; 56(3)1510–1521. doi: 10.1021/acs.est.1c04158 35038861 PMC8811958

