# Supplementary material for: Vibrio spp and other potential pathogenic bacteria associated to microfibers in the North-Western Mediterranean Sea
Source: PLoS One. 2022 Nov 30;17(11):e0275284. doi: 10.1371/journal.pone.0275284 (PMC9710791; doi:10.1371/journal.pone.0275284)
Supplement: S1 File — (DOCX) [file pone.0275284.s001.docx]

The following documents are available online at <https://doi.org/10.6084/m9.figshare.20227332>: (1) “MFs Microbiome_FAS file.fas” with the DNA sequences, (2) "MFs microbiome_OTU table and Statistics" with the taxonomy and number of reads per sample, as well as the Chao estimates, (3) a link to access the Krona platform for graphical visualization of microbial diversity on MFs per sample, (4) Microphotograph of visually identified microfibers (length in µm).
